# Association between social support and postpartum depression

**DOI:** 10.1038/s41598-022-07248-7

**Published:** 2022-02-24

**Authors:** Hahyeon Cho, Kyeongmin Lee, Eunji Choi, Ha Na Cho, Boyoung Park, Mina Suh, Yumie Rhee, Kui Son Choi

**Affiliations:** 1 National Cancer Center, Graduate School of Cancer Science and Policy, 323 Ilsan-ro, Ilsandong-gu, Goyang, Gyeonggi-do 10408 Republic of Korea; 2 Quantitative Sciences Unit, Stanford University School of Medicine, Stanford, CA USA; 3 Department of Medicine, Hanyang University College of Medicine, Seoul, Republic of Korea; 4 National Cancer Control Institute, National Cancer Center, Goyang-si, Republic of Korea; 5 Department of Internal Medicine, Endocrine Research Institute, Severance Hospital, Yonsei University College of Medicine, Seoul, Republic of Korea

**Keywords:** Health care, Risk factors

## Abstract

Postpartum depression is common; however, little is known about its relationship to social support and postpartum depression. This study examined the association between them among South Korean women within one year of childbirth. This study was based on the 2016 Korean Study of Women’s Health-Related Issues (K-Stori), a cross-sectional survey employing nationally-representative random sampling. Participants were 1,654 postpartum women within a year of giving birth. Chi-square test and logistic regression analysis were conducted to analyze the associations between social support (and other covariates) and postpartum depression. Among participants, 266 (16.1%) had postpartum depression. Depending on the level of social support, 6.0%, 53.9%, and 40.1% of them had low, moderate, and high social support, respectively. Women with moderate or low social support were more likely to have postpartum depression (OR = 1.78, 95% CI = 1.26–2.53; OR = 2.76, 95% CI = 1.56–4.89). This trend was observed in participants with multiparity, pregnancy loss, obese body image, and employed women. Social support was associated with a decreased likelihood of postpartum depression, indicating the importance of social support, especially for women experiencing multiparity, pregnancy loss, negative body image, as well as for employed women.

## Introduction

One of the most important turning points in a woman’s life is pregnancy and childbirth^1^. Women at the prenatal and postpartum periods commonly experience mental health problems^2^. The onset of mental health problems can cause devastation and dissension in a woman’s life^3^. Depression at the prenatal and postpartum periods is a common and debilitating psychiatric disorder, prevalent in South Korea and worldwide^4,5^. Incidence of depression is approximately two times higher in women than in men, and is particularly common among women of child-bearing age^6^. In Korea, social burden due to mental disorders have significantly increased. The proportion of those who suffered from mental disorders at least once in their lifetime was 30.2% in 2006 and 27.6% in 2011^7^. The consequences of depression during the postpartum period are considerably more deleterious because a woman faces the added responsibility of being a caregiver for her newborn infant^3^.

Postpartum depression (PPD) is the most common complication of childbearing, affecting approximately 10–22% of woman in childbearing age^8^. Women usually experience PPD within the first six weeks following delivery, and recover from it six months postpartum; however, it may continue through the first and second years postpartum^9^. PPD is closely linked with negative outcomes for women and infants, such as maternal suicide, weak maternal interaction with her infant, early termination of breastfeeding, and delay in the child’s development^10,11^. Previous studies identified the following risks factors as predictors of PPD: low social support^2,10,12^, prenatal depression^13,14^, prenatal anxiety^15^, pregnancy loss experience^16,17^, perceived body image^15,18,19^, child care stress^20^, parity^21–23^, marital relationship^24^, socioeconomic status^13^, and currently employed status^25–27^.

Although PPD is undetected or inadequately treated, it may be remedied by both, behavioral and pharmacological treatments^28^. However, pharmacological therapy was unsuitable for postpartum mothers, particularly those who wished to breastfeed, as antidepressant compounds may be passed onto the infant via breastmilk^29,30^. Thus, prevention is the best method against the onset of depression rather than relying on these treatments. While the cause of PPD remains unclear, social support has been shown to be effective in helping women cope with postpartum depression^12,31^. Social support is defined as an exchange of resources among people perceived by the provider or recipient to be intended to improve the lifestyle of the recipient^32^. Social support includes both practical and emotional supports that are provided to an individual through social network. Sources of such supports are the individual’s family, friends, or significant others^33^. Social support can be measured in the form of perceived social support, which subjectively measures an individual's beliefs about the available support. It reflects an individuals' feeling that they are accepted, loved, and valued by other members of their social network^34,35^.

Previous studies have investigated the relationship between PPD and social support^2,10,12^ However, there was relatively little consideration for factors affecting social support and postpartum depression. Therefore, the aim of this study was to estimate the prevalence of PPD in postpartum women in South Korea, and investigate the relationship between PPD and social support.

## Results

A total of 1,654 postpartum women were included in the analysis, of which 266 (16.1%) had PPD and 1,388 (83.9%) did not. The demographic and health characteristics of the study participants are summarised in Table 1. Statistically significant differences were observed between social support and having PPD. There were a greater number of women with PPD in groups with low social support.

**Table 1:** General characteristics of the study population afflicted by postpartum depression in this study.

Variables		Postpartum depression						*P*-value
		Total		Yes		No		
		N	%	N	%	N	%	
Total		1,654	100.0	266	16.1	1,388	83.9	
Social support^a^	High	663	40.1	55	14.3	608	4.4	** < .0001**
	Moderate	892	53.9	173	65.0	719	51.8	
	Low	99	6.0	38	20.7	61	43.8	
Age (in years)	19–29	255	15.4	41	15.4	214	15.4	0.5318
	30–39	1,231	74.4	203	76.3	1,028	74.1	
	40–44	168	10.2	22	8.3	146	10.5	
Region	Urban area	1,347	81.4	217	81.6	1,130	81.4	0.9489
	Rural area	307	18.6	49	18.4	258	18.6	
Education level	≤ High school	387	23.4	79	22.2	308	29.7	**0.008**
	≥ College	1,267	76.6	187	77.8	1,080	70.3	
Household income	< 3,000$/month	417	25.2	92	34.6	325	23.4	**0.0005**
	3,000–4,999$/month	1,064	64.3	152	57.1	912	65.7	
	≥ 5,000$/month	173	10.46	22	8.3	151	10.9	
Current job	No	1,255	75.9	163	61.3	1,092	78.7	** < .0001**
	Yes	399	24.1	103	38.7	163	21.3	
Parity	Primipara	645	39.0	98	36.8	547	39.4	0.4317
	Multipara	1,009	61.0	168	63.2	841	60.6	
Pregnancy loss experience	None	1,301	78.7	212	79.7	1,089	78.5	0.6509
	Had	353	21.3	54	20.3	299	21.5	
Current breastfeeding	Yes	866	52.4	116	43.6	750	54.0	**0.0018**
	No	788	47.6	150	56.4	638	46.0	
Degree of parenting burden within the last month	Low	363	21.9	11	4.1	352	25.4	0.1926
	Moderate	949	57.4	159	59.8	790	56.9	
	High	342	20.7	96	36.1	246	17.7	
Subjective health status	High	1,162	70.2	140	52.6	1,022	73.6	** < .0001**
	Moderate	435	26.3	94	35.4	341	24.6	
	Low	57	3.5	32	12.0	25	1.8	
Perceived body image	Underweight	86	5.2	19	7.1	67	4.8	**0.0114**
	Normal	885	53.5	121	45.5	764	55.1	
	Obese	683	41.3	126	47.4	557	40.1	
Stress level	Low	292	17.7	17	6.4	275	19.8	** < .0001**
	Moderate	1,136	68.7	168	63.2	968	69.7	
	High	226	13.6	81	30.4	145	10.5	
Past diagnosis of depression	None	1,611	97.4	26	60.5	1,362	84.5	** < .0001**
	Had	43	2.6	17	39.5	249	15.5	
Drinking alcohol during pregnancy	None	1,252	75.7	193	72.6	1,059	76.3	0.1926
	Had	402	24.3	73	27.4	329	23.7	
Smoking experience	None	1,596	96.5	248	93.2	1,348	97.1	**0.0016**
	Had	58	3.5	18	6.8	40	2.9	

^a^Social support: Total Multidimensional Scale of Perceived Social Support (MSPSS) score range from 12–24 (low), 25–36(moderate), 37–48 (high).

Boldface indicates statistical significance (*p* < 0.05).

Table 2 shows the social support status among participants: 6.0% of women had low, 53.9% had moderate and 40.1% had high social support. Overall, women who responded that social support was low had a low level of education and income, were employed, and faced high parenting burden during the previous month (*p* < 0.001). In addition, women with lower level of social support had relatively higher level of stress, with a history of depression and smoking (*p* < 0.001). Meanwhile, women with high social support had high breastfeeding percentages and high level of subjective health status (*p* < 0.001). Univariate and multiple logistic regression were conducted to determine the association between PPD and social support while controlling for potential covariates (Table 3). In univariate logistic regression analyses, the women with moderate (OR = 2.66, 95% CI = 1.93–3.67) and low (OR = 6.89, 95% CI = 4.22–11.24) level of social supports had increased likelihood for PPD compared to the women with high level of social support. In multivariate logistic regression analyses, the women with moderate and low social support levels were 1.78 (95% CI = 1.25–2.52) and 2.73 (95% CI = 1.54–4.83) times more likely to develop PPD, respectively, as compared to the women with high social support levels. Further, women who were employed, breastfed, faced high parenting burdens during the previous month, had poor subjective health status, high stress level, and a past depression diagnosis were more likely to develop PPD.

**Table 2:** General characteristics of the study population by social support in this study.

Variables		Social support								*P*-value
		Total		Low		Moderate		High		
		N	%	N	%	N	%	N	%	
Total		1,654	100.0	99	6.0	892	53.9	663	40.1	
Age (in years)	19–29	255	15.4	17	17.2	136	15.3	102	15.4	0.6739
	30–39	1,231	74.4	68	68.7	667	74.8	496	74.8	
	40–44	168	10.2	14	14.1	89	9.9	65	9.8	
Region	Urban area	1,347	81.4	76	76.8	735	82.4	536	80.8	0.3451
	Rural area	307	18.6	23	23.2	157	17.6	127	19.2	
Education level	≤ High school	387	23.4	36	36.4	224	25.1	127	19.2	**0.0002**
	≥ College	1,267	76.6	63	63.6	668	74.9	536	80.8	
Household income	< 3,000$/month	417	25.2	48	48.5	238	26.7	131	19.8	** < .0001**
	3,000–4,999$/month	1,064	64.3	46	46.5	569	63.8	449	67.7	
	≥ 5,000$/month	173	10.5	5	5.0	85	9.5	83	12.5	
Current job	No	1,255	75.9	77	77.8	645	72.3	553	80.4	**0.001**
	Yes	399	24.1	22	22.2	247	27.7	130	19.6	
Parity	Primipara	645	39.0	47	47.5	323	36.2	275	41.5	0.4317
	Multipara	1,009	61.0	52	52.5	569	63.8	388	58.5	
Pregnancy loss experience	None	1,301	78.7	83	83.8	718	80.5	500	75.4	**0.0232**
	Had	353	21.3	16	16.2	174	19.5	163	24.6	
Current breastfeeding status	Yes	866	52.4	44	44.4	460	51.6	362	54.6	**0.0018**
	No	788	47.6	55	55.6	432	48.4	301	45.4	
Degree of parenting burden within the last month	Low	363	21.9	6	6.1	149	16.7	208	31.4	** < .0001**
	Moderate	949	57.4	64	64.6	532	59.6	353	53.2	
	High	342	20.7	29	29.3	211	23.7	102	15.4	
Subjective health status	High	1,162	70.2	46	46.5	614	68.8	502	75.7	** < .0001**
	Moderate	435	26.3	42	42.4	242	27.1	151	22.8	
	Low	57	3.5	11	11.1	36	4.1	10	1.5	
Perceived body image	Underweight	86	5.2	6	6.1	56	6.3	24	3.6	0.2281
	Normal	885	53.5	52	52.5	471	52.8	362	54.6	
	Obese	683	41.3	41	41.4	365	40.9	277	41.8	
Stress level	Low	292	17.7	11	11.1	154	17.3	127	19.1	** < .0001**
	Moderate	1,136	68.7	50	50.5	618	69.3	468	70.6	
	High	226	13.6	38	38.4	120	13.4	68	10.3	
Past diagnosis of depression	None	1,611	97.4	93	93.9	864	96.9	654	98.6	**0.0076**
	Had	43	2.6	6	6.1	28	3.1	9	1.4	
Smoking experience	None	1,596	96.5	91	91.9	858	96.2	647	97.6	
	Had	58	3.5	8	8.1	34	3.8	16	2.4	**0.0128**

Boldface indicates statistical significance (*p* < 0.05).

**Table 3:** Univariable and multivariable logistic regression analyses of the relationship between social support and postpartum depression.

Variables		Postpartum depression			
		Univariable logistic regression		Multivariable logistic regression	
		cOR	95% CI	aOR	95% CI
Social support	High	1.00		1.00	
	Moderate	2.66	1.93–3.67	1.78	1.25–2.52
	Low	6.89	4.22–11.24	2.73	1.54–4.83
Age (in years)	19–29	1.00		1.00	
	30–39	1.03	0.71–1.49	1.03	0.67–1.57
	40–49	0.79	0.45–1.38	0.77	0.40–1.45
Region	Urban area	1.00		1.00	
	Rural area	0.99	0.71–1.39	0.98	0.67–1.43
Education level	≤ High school	1.00		1.00	
	≥ College	0.68	0.50–0.90	0.77	0.54–1.08
Household income	< 3,000$/month	1.00		1.00	
	3,000–4,999$/month	0.59	0.44–0.79	0.76	0.54–1.07
	≥ 5,000$/month	0.52	0.31–0.85	0.50	0.27–0.90
Current job	No	1.00		1.00	
	Yes	2.33	1.77–3.08	2.80	2.02–3.88
Parity	Primipara	1.00		1.00	
	Multipara	1.12	0.85–1.46	0.82	0.58–1.16
Pregnancy loss experience	None	1.00		1.00	
	Had	0.93	0.67–1.28	1.01	0.68–1.51
Current breastfeeding status	Yes	1.00		1.00	
	No	1.52	1.17–1.98	1.48	1.10–2.01
Degree of parenting burden within the last month	Low	1.00		1.00	
	Moderate	6.44	3.45–12.01	5.10	2.66–9.98
	High	12.48	6.55–23.79	10.09	5.08–20.04
Subjective health status	High	1.00		1.00	
	Moderate	2.01	1.51–2.69	1.48	1.07–2.05
	Low	9.34	5.38–16.23	5.41	2.83–10.36
Perceived body image	Underweight	1.00		1.00	
	Normal	0.56	0.32–0.96	0.98	0.52–1.87
	Obese	0.80	0.46–1.38	1.24	0.65–2.36
Stress level	Low	1.00		1.00	
	Moderate	2.81	1.68–4.71	2.28	1.32–3.95
	High	9.04	5.16–15.82	4.76	2.58–8.79
Past diagnosis of depression	None	1.00		1.00	
	Had	3.58	1.91–6.69	2.78	1.34–5.78
Smoking experience	None	1.00		1.00	
	Had	2.45	1.38–4.34	1.69	0.83–3.44

The results of subgroup analyses on social support and PPD with covariates are summarised in Table 4. Among women with multiparity, those with moderate and low levels of social support were 2.85 (95% CI = 1.68–4.82) and 4.90 (95% CI = 2.14–11.23) times more likely to develop PPD, respectively; but there were no statistical differences in women with primiparity. Further, in women who experienced pregnancy loss, those with lower levels of social support were 10.26 times more likely to develop PPD compared to women receiving high levels of social support. Among women who reported their body image as normal or obese, women with moderate and low levels of social support were more likely to develop PPD. In addition, women with jobs and low levels of social support showed the highest likelihood of PPD (OR = 10.34, 95% CI = 2.34–45.64).

**Table 4:** The results of subgroup analysis of postpartum depression to social support stratified by parity, pregnancy loss experience perceived body image, current employment status.

Variables		Postpartum depression					*P*-value For trend
		Social support					
		High	Moderate		Low		
		OR	aOR	95% CI	aOR	95% CI	
Parity	Primipara	1.00	1.05	0.62–1.78	1.74	0.74–4.10	0.0017
	Multipara	1.00	2.85	1.68–4.82	4.90	2.14–11.23	< .0001
Pregnancy loss experience	None	1.00	1.58	1.07–2.35	2.15	1.14–4.05	< .0001
	Had	1.00	2.36	0.99–5.63	10.26	2.16–48.73	< .0001
Perceived body image	Underweight	1.00	0.44	0.03–5.81	0.52	0.02–13.07	0.0987
	Normal	1.00	2.45	1.46–4.10	3.35	1.43–7.85	< .0001
	Obese	1.00	1.70	1.02–2.83	2.39	1.05–5.41	< .0001
Currently employed	No	1.00	1.97	1.27–3.05	2.39	1.21–4.71	< .0001
	Yes	1.00	1.76	0.93–3.34	10.34	2.34–45.64	< .0001

**Figure 1:**
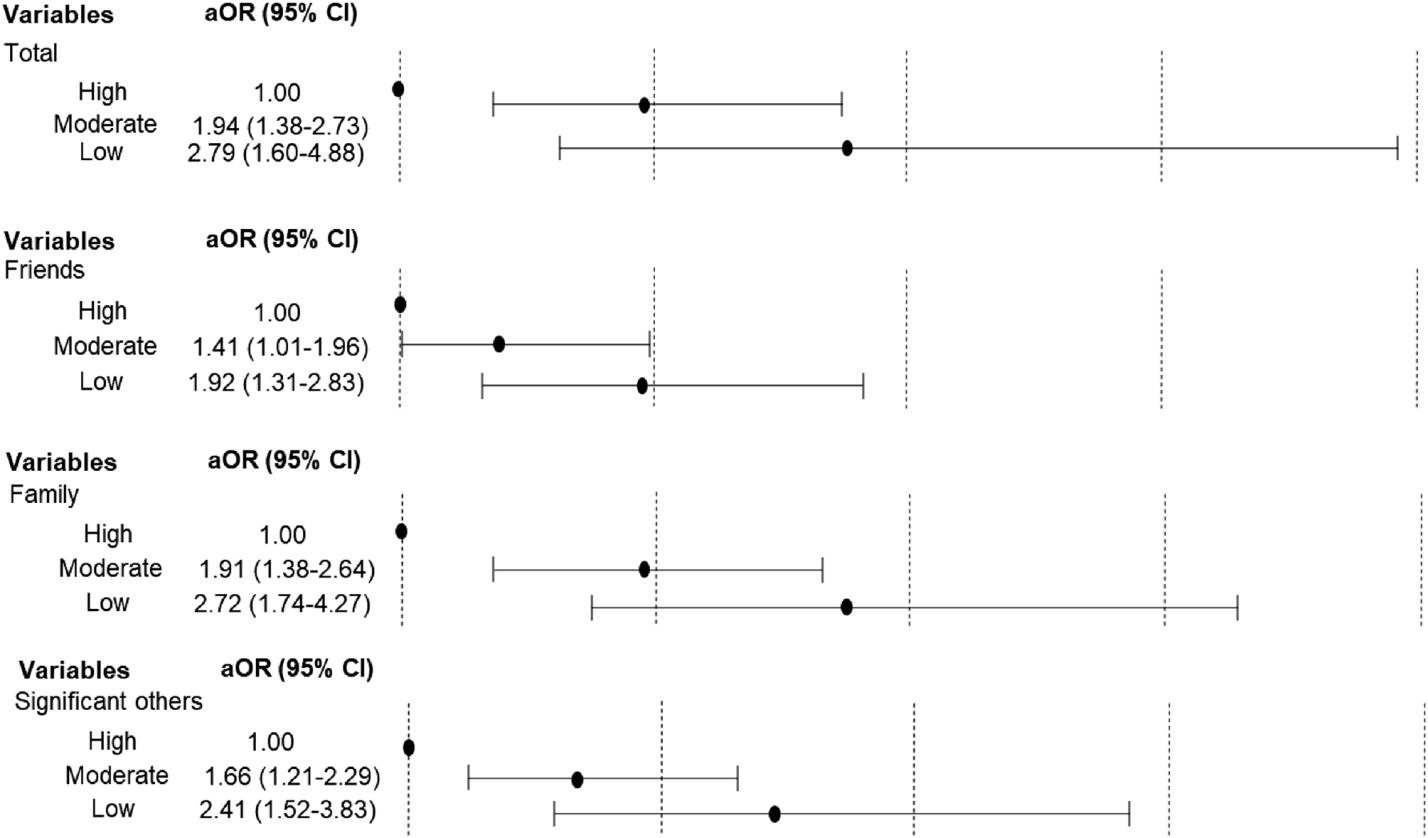
The results of subgroup analysis based on postpartum depression according to social support by social support subscale.

Figure 1 presents the relationship between the level of social support in each scale of the development of PPD, and shows that the lower the social support level in all subscales, the higher the odds ratios (ORs) of PPD. The results showed a high degree of association in order of family, significant others, and friends.

## Discussion

The World Health Organization (WHO) noted that mental health problems such as depression and anxiety are common during pregnancy and after childbirth^26^. However, there was limited information on which women develop PPD. Therefore, this study was conducted to investigate the associated factors of PPD, especially how social support affects depression in postpartum women. Our findings show that postpartum women with low social support had 4.63-fold higher odds of PPD compared with postpartum women with high social support. Further, women who are employed or breastfeeding, or have heavy parenting burden, poor subjective health status, high stress level, and were diagnosed with depression in the past were more likely to develop PPD. Furthermore, this study also showed that higher levels of social support may buffer against probability of PPD after adjusting for confounding variables. Given our results, postpartum women need a high level of social support from family, close friends, and significant others.

Interestingly, the subgroup analysis showed that women with multiparity had a five times higher risk of PPD if their social support was low. Multiparity could increase the level of maternal stress and depression because women need to also care for their previous children and infants. Women with multiparity may not receive the same level of social support as they received during their first time of childbearing because they are considered to be child-care experts despite their need of extra social support to take care of their new-born babies. Thus, the results suggest that as women with multiparity are more likely to have PPD, better social support is needed for preventing PPD^27^.

Another interesting finding in the current study is that women with low social support and previous experience of pregnancy loss were 10 times more likely to develop PPD. Our result is in line with previous studies in which previous pregnancy loss served as an effect modifier between social support and PPD. Pregnancy loss is an event which makes bereaved women particularly prone to depression, mood disorders, dramatic mental health disorder, and even suicide^36^. According to previous research, women who have lost their babies were seven to nine times more vulnerable to depression than women without a pregnancy loss^37^. These results indicate that postpartum women with history of pregnancy loss need higher level of social support to prevent PPD.

Body image also acts as an effect modifier between social support and PPD. Similar to adolescence, the period surrounding childbirth is accompanied by unique and rapid changes in not only body shape and size, but also psychological dimensions. Prior studies suggested that about 85% of women during pregnancy experience body image dissatisfaction^38^. In recent years, an increasing number of women reported to be concerned about their weight gain and appearance during pregnancy and postpartum period^39,40^. Body image may influence depression and health behaviours in postpartum women^41^. The current study results suggest that social support is an important factor that improves mental health of women with negative body image during postpartum period.

Several countries have implemented paid leave to help working parents^42^. Despite policies like paid leaves, women continue working for reasons such as career, being worried about losing their jobs, financial burden, and negative attitudes in the workplace. Women who have to multitask by having a job and having to rear child are especially vulnerable PPD^43^. Similarly, the current study results showed that employed women were 2.8 times more likely to develop PPD. Job strain often cannot be decreased or relieved, but social support in workplace can buffer the negative effect of overwork and role ambiguity^25,43^. In addition to workplace support, social support from their partner, family, and friends also decrease job stress in postpartum women^44^.

The strength of the study is that the findings are based on a nationwide survey. This ensures that the data is representative of Korean women, and comprehensively assesses depressive symptoms of the postpartum period.

## Limitations

Several limitations to the present study could influence the interpretation of our findings. First, the cross-sectional design of K-stori could not show the direction of the causal relationship for the identified association between social support and PPD. Second, this study primarily relied on self-report measures from K-stori. Thus, questions on the dependent and other independent variables may contribute to recall bias. Third, depression was measured based on a self-report. Previous research has shown that self-reported survey can be under or overestimated depending on individual characteristics. Though all the responses were anonymous, quite a few individuals may not indicate their true levels of depression^45^.

This study provided a cross-sectional estimate of PPD within one year of childbirth in South Korean women. Social support was also positively associated with a lower likelihood of PPD in women with multiparity, pregnancy loss experience, negative body image, and jobs. Postpartum women should receive a high level of social support from family, friends, and significant others to prevent PPD and improve their maternal health, aided by health professionals.

## Method

### Study population

This cross-sectional study was based on the Korean Study of Women’s Health-Related Issues (K-Stori) in 2016. It was approved by the Institutional Review Board of the National Cancer Center, Korea (Approval no: NCC2016-0062). The K-Stori is a nationwide survey designed to investigate a broad area of health issues among Korean women according to five stages in the life cycle of a woman (adolescence: 14–17 years; childbearing: 19–44 years; pregnancy and postpartum: 19–44 years; menopause: 45–64 years; and old adulthood: 65–79 years)^46^. An approved study description was provided to all eligible participants. The study description covered the research purpose, subject, content, duration, voluntary participation, withdrawal of consent, expected risks and benefits from participating in the research, publication of the study results, and confidentiality. If the subjects agreed to participate in the study after reading the study description, participants were asked to provide written informed consent^46^. All methods were carried out in accordance with approved guidelines and regulations.

The participants of this study were women within a year of giving birth. From the total pregnant and postpartum women (n = 3000), pregnant women (n = 1,346) were excluded, and thus 1,654 women aged 19–44 years were included in the study. To recruit the participants, the interviewers planned to visit obstetrics and gynecology, or post-partum care centers. In order to select the same survey area as the other life cycle stages, a systematic data extraction method was used to identify local obstetrician and post-partum care centers, for pregnant and post-partum women, based on the same sample design area for the household survey participants.

### Measures

The main outcome variable of this study was PPD, which was evaluated using the Edinburgh Postnatal Depression Scale (EPDS)^47^. The EPDS is a validated 10 questions screening tool, and is the most widely used screening questionnaire asking mothers how they have felt in the past seven days for PPD (Appendix 1). Participants answered the questionnaires and the answers were scored from 0–3 points (or 3–0 in case of a reverse score) with a total score of 0 to 30. A threshold score of ≥ 10 was used to classify postpartum women with a probable major depression who needed further medical examination based on the Korean version of the EPDS^13,48,49^.

The Multidimensional Scale of Perceived Social Support (MSPSS) was used to measure individual perceived social supports from three sources: friends (Item 6, 7, 9, and 12), family (Item 3, 4, 8, and 11) and significant others (Item 1, 2, 5, and 10)^50^. Participants were asked to indicate their agreement with the statements on a five-point Likert scale ranging from 0 = very strongly disagree to 4 = very strongly agree. The total score was calculated as the mean of 12 scores. The subscale total scores were the sum of the scores for the four questions related to the subscale. Total scores ranging from 12 to 24 were classified as low social support, from 25 to 36 as moderate social support and from 37 to 48 as high social support.

Further, information on age, living area, education level, household income, current job, number of parities, pregnancy loss experience, current breastfeeding status, degree of parenting burden within the last month, subjective health status, perceived body image, stress, past diagnosis of depression, and smoking experience was collected.

### Statistical analyses

Descriptive analysis was conducted to compare the characteristics of study participants according to postpartum depression level. Multiple logistic regression was used to determine the association between PPD and social support while controlling for potential covariates. The OR and 95% confidence interval (95% CI) of having PPD were estimated.* P* values < 0.05 were considered statistically significant. Furthermore, subgroup analyses were conducted to assess the influence of social support on the risk of having PPD according to groups with different characteristics. In the subgroup analyses, the Cochran-Armitage test was used to assess the association between PPD and each variable, and the awareness of nutrition labelling. *P* values < 0.05 were considered to be statistically significant. All statistical analyses were performed using SAS version 9.4 (Cary, NC, USA).

## Supplementary Information

Supplementary Information.
