# Author Correction: Association between social support and postpartum depression

**DOI:** 10.1038/s41598-022-08119-x

**Published:** 2022-03-04

**Authors:** Hahyeon Cho, Kyeongmin Lee, Eunji Choi, Ha Na Cho, Boyoung Park, Mina Suh, Yumie Rhee, Kui Son Choi

**Affiliations:** 1 National Cancer Center, Graduate School of Cancer Science and Policy, 323 Ilsan‑ro, Ilsandong‑gu, Goyang, Gyeonggi‑do 10408 Republic of Korea; 2 Quantitative Sciences Unit, Stanford University School of Medicine, Stanford, CA USA; 3 Department of Medicine, Hanyang University College of Medicine, Seoul, Republic of Korea; 4 National Cancer Control Institute, National Cancer Center, Goyang‑si, Republic of Korea; 5 Department of Internal Medicine, Endocrine Research Institute, Severance Hospital, Yonsei University College of Medicine, Seoul, Republic of Korea

Correction to: *Scientific Reports* 10.1038/s41598-022-07248-7, published online 24 February 2022

The original version of this Article contained an error in the Acknowledgments section.

“This study was funded by the Korea Center for Disease Control and Prevention (Grant number: 2015ER630300), and a Grant-in-Aid for Cancer Research and Control from the National Cancer Center, Korea (#1910231).”

now reads:

“This study was funded by the Korea Center for Disease Control and Prevention (Grant number: 2015ER630300), and a Grant-in-Aid for Cancer Research and Control from the National Cancer Center, Korea (#2210772).”

The original Article has been corrected.

