# Supplementary material for: Association between social support and postpartum depression
Source: Sci Rep. 2022 Feb 24;12:3128. doi: 10.1038/s41598-022-07248-7 (PMC8873474; doi:10.1038/s41598-022-07248-7)
Supplement: Supplementary file 1 — Supplementary Information. [file 41598_2022_7248_MOESM1_ESM.docx]

**Appendix 1. Edinburgh Postnatal Depression Scale (EPDS)**

Please check the answer which comes closest to how you have felt in the past 7 days.

**Q1. I have been able to laugh and see the funny side of things**

0. As much as I always could

1. Not quite so much now

2. Definitely not so much now

3. Not at all

**Q2. I have looked forward with enjoyment to things**

0. As much as I ever did

1. Rather less than I used to

2. Definitely less than I used to

3. Hardly at all

**Q3. I have blamed myself unnecessarily when things went wrong**

0. Yes, most of the time

1. Yes, some of the time

2. Not very often

3. No, never

**Q4. I have been anxious or worried for no good reason**

0. No, not at all

1. Hardly ever

2. Yes, sometimes

3. Yes, very often

**Q5. I have felt scared or panicky for no very good reason**

0. Yes, quite a lot

1. Yes, sometimes

2. No, not much

3. No, not at all

**Q6. Things have been getting on top of me**

0. Yes, most of the time I haven't been able to cope at all

1. Yes, sometimes I haven't been coping as well as usual

2. No, most of the time I have coped quite well

3. No, I have been coping as well as ever

**Q7. I have been so unhappy that I have had difficulty sleeping**

0. Yes, most of the time

1. Yes, sometimes

2. Not very often

3. No, not at all

**Q8. I have felt sad or miserable**

0. Yes, most of the time

1. Yes, quite often

2. Not very often

3. No, not at all

**Q9. I have been so unhappy that I have been crying**

0. Yes, most of the time

1. Yes, quite often

2. Only occasionally

3. No, never

**Q10. The thought of harming myself has occurred to me**

0. Yes, quite often

1. Sometimes

2. Hardly ever

3. Never
